# Supplementary material for: The Configuration of the Perivascular System Transporting Macromolecules in the CNS
Source: Front Neurosci. 2019 May 28;13:511. doi: 10.3389/fnins.2019.00511 (PMC6547014; doi:10.3389/fnins.2019.00511)
Supplement: Supplementary file 1 [file Table_1.DOC]

Supplemental Materials


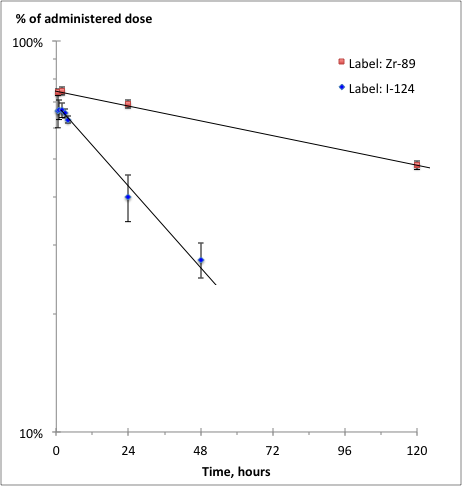


Figure S1. Lysosomal stability of rhNAGLU in vivo (rats).

Label clearance from the liver after intravenous administration of rhNAGLU labeled with ^124^I and ^89^Zr (N=3 each), PET/CT, ROI: whole liver.

Clearance of ^124^I (t_1/2_=33±3 hours) reflects the rate of lysosomal fragmentation followed by rapid deiodination of the fragments and immediate release of [^124^I]-iodide to the blood. The estimated fraction of intact (non-fragmented) rhNAGLU24 hours after uptake by cells is 60±10%.


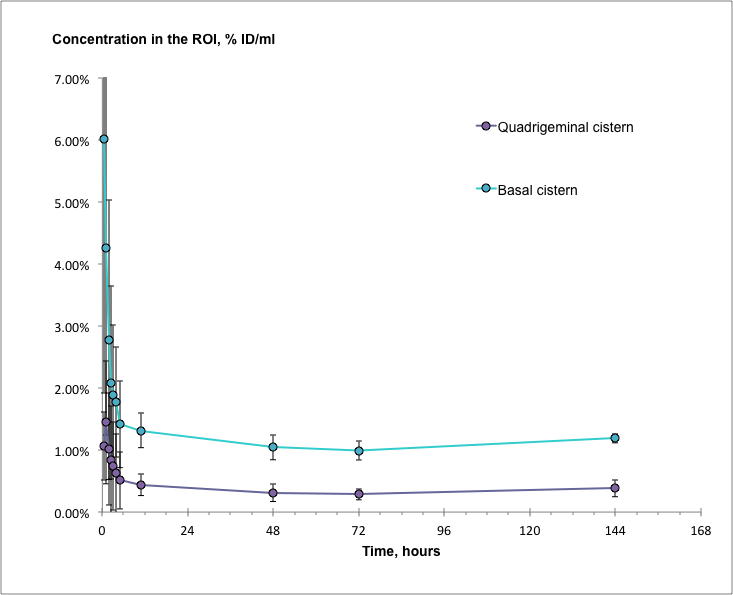


Figure S2. - Clearance of [^89^Zr]rhNAGLU from the CSF of monkeys (N=4), PET/CT data. The persisting fraction reflects protein deposition in the endocytosing cells of the cistern boundaries. By 24 hours, rhNAGLU is practically cleared from the CSF (estimated t_1/2_=2±0.5 hours, clearance by 24 hours >99.8%). Clearance from the cisternal CSF of rat was not measured due to insufficient resolution.


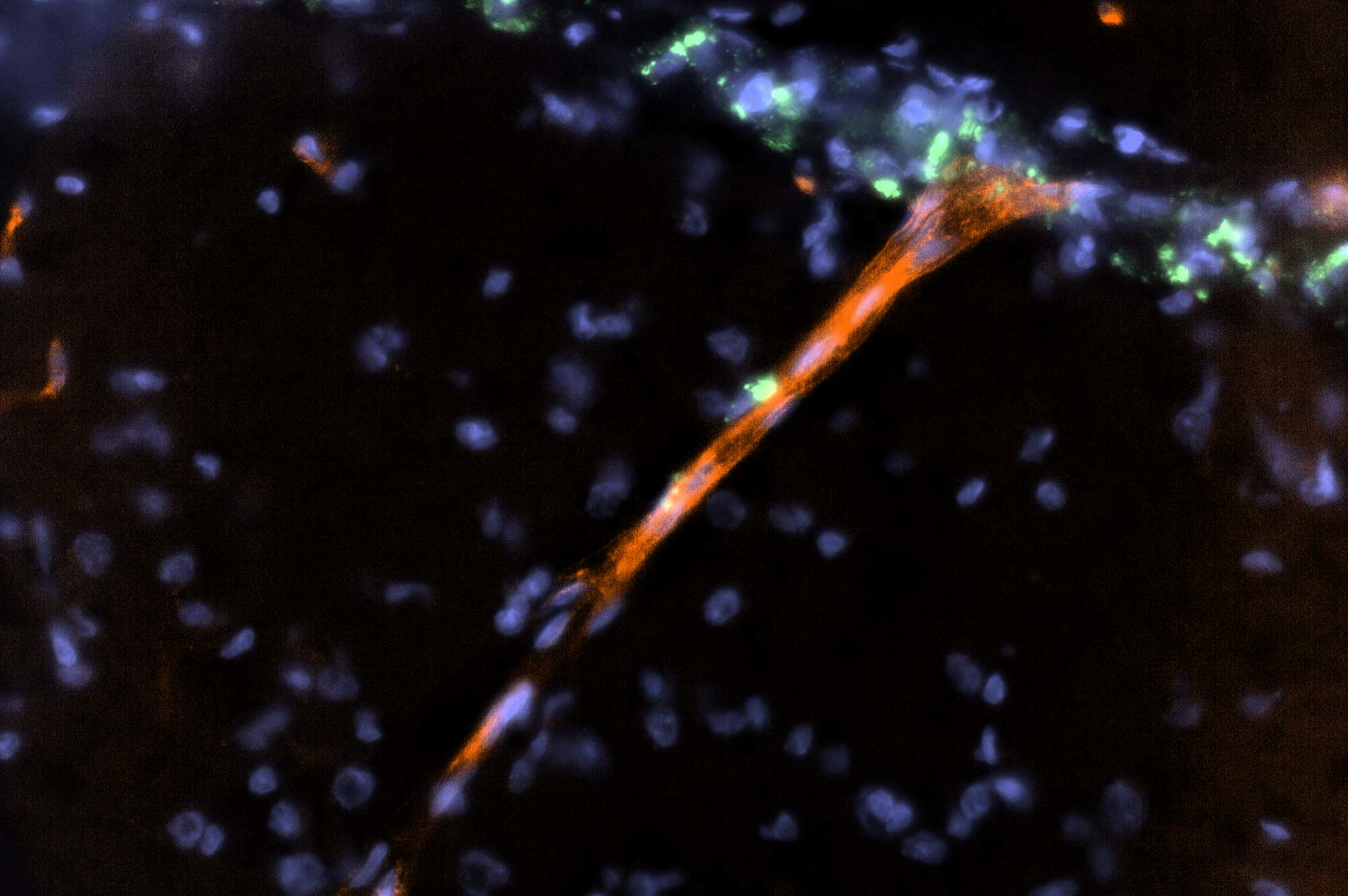


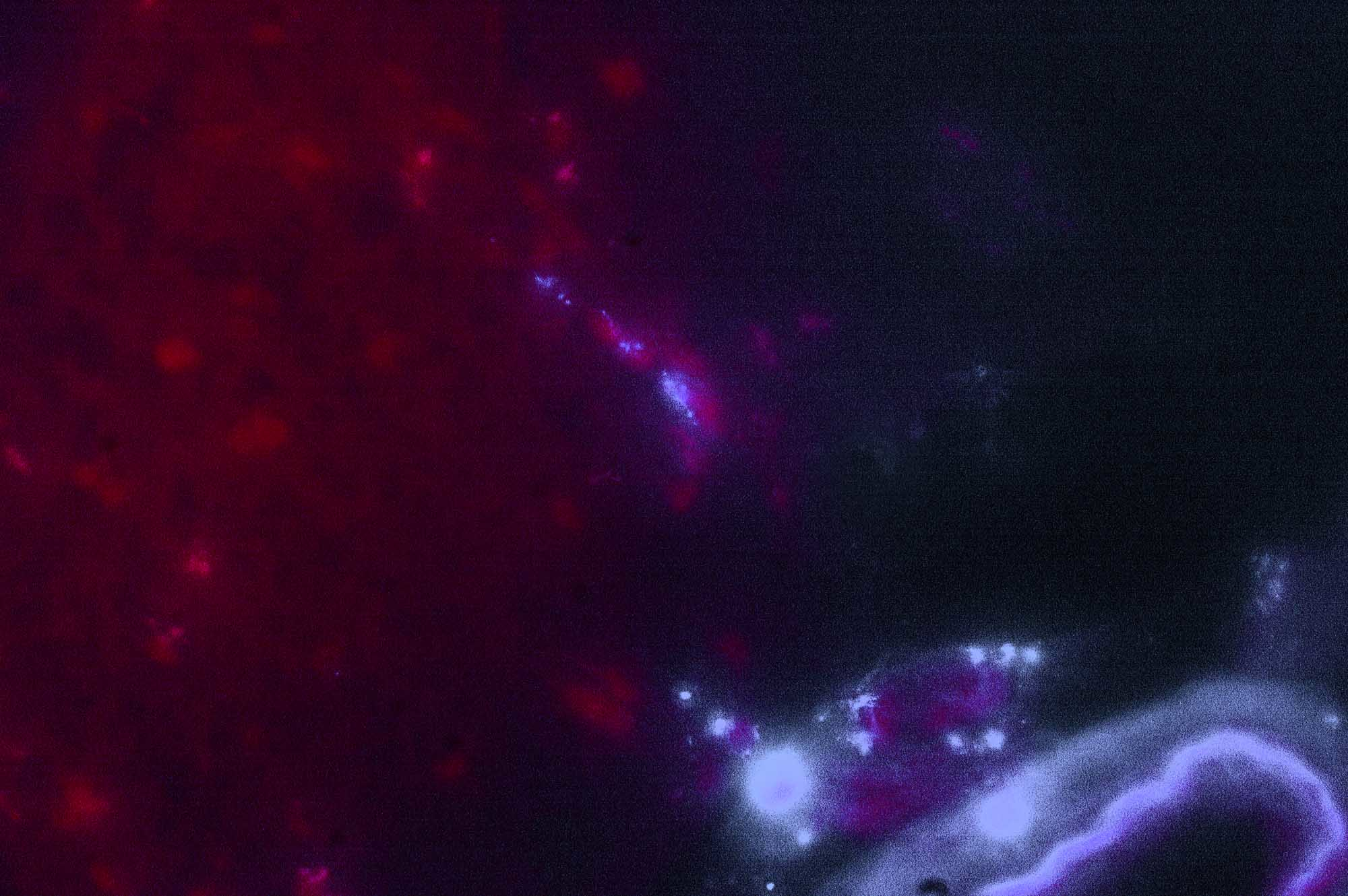


Figure S3. Perivascular cells (orange arrows) labeled with hrNAGLU.

Top: rat, blood vessel (red) branching from the subcortical fissure (F); vesicular accumulation of FITC-hrNAGLU (green), nuclear stain (cyan).

Bottom: monkey, blood vessel (unstained) in bregma; vesicular accumulation of Alexa 350-hrNAGLU (cyan), nuclear stain (red)


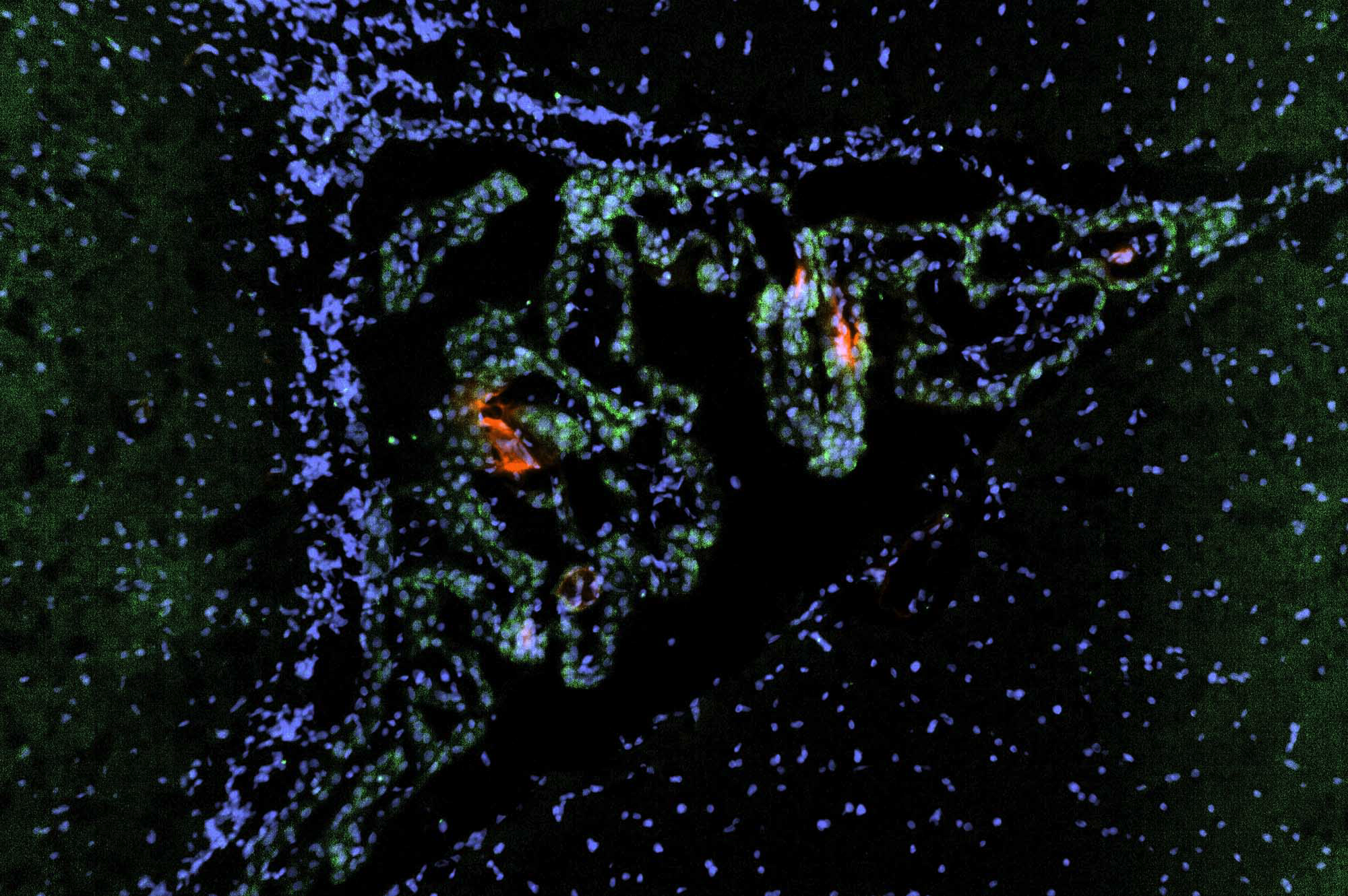


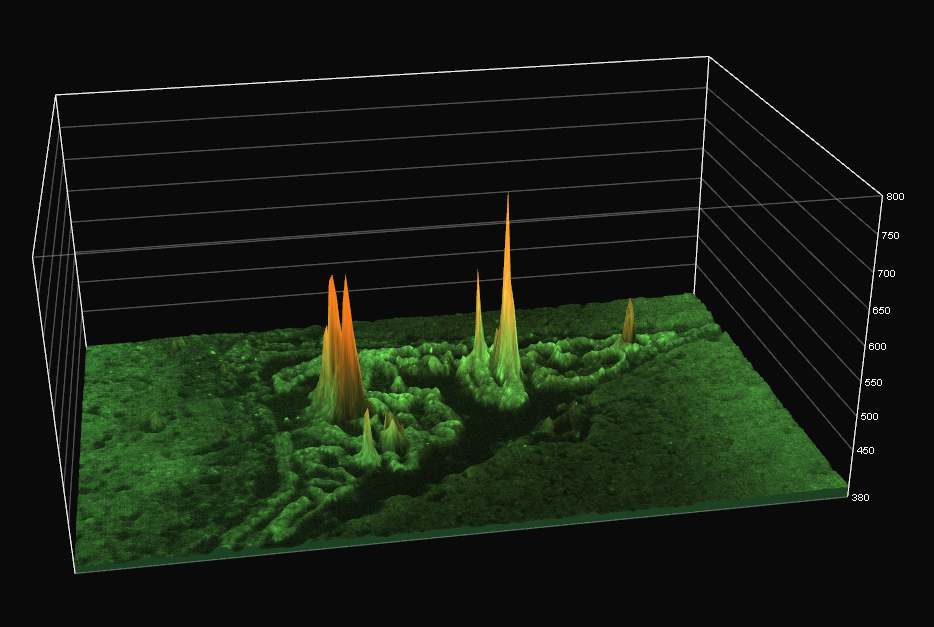


Figure S4. Lateral ventricle, rat.

Top: 20 μm slice, 0.9X0.6 mm. Red: blood vessels. Green: FITC-hrNAGLU. Cyan: nuclear stain.

Bottom: Surface intensity plot for the above image (nuclear stain not shown).


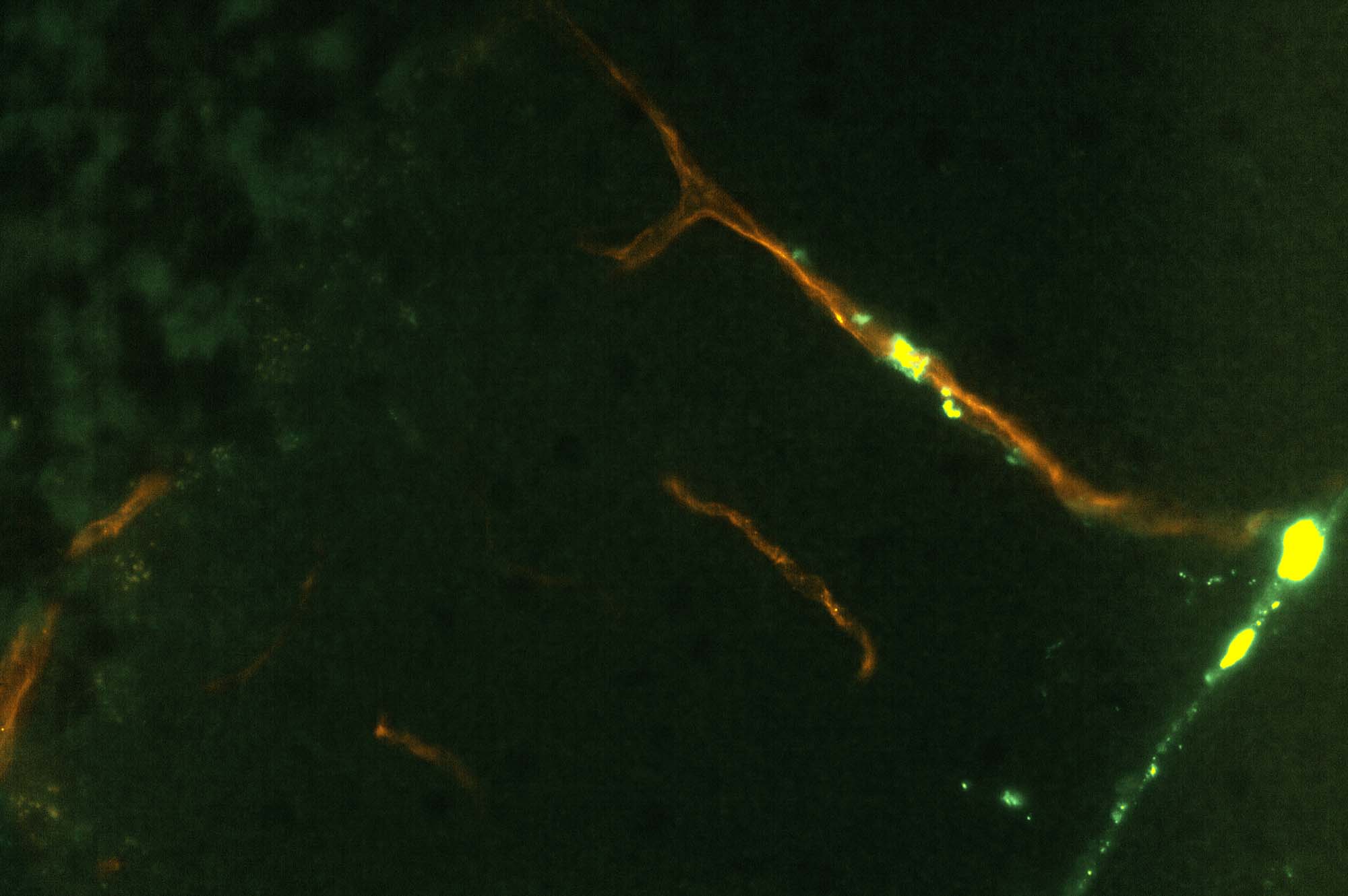


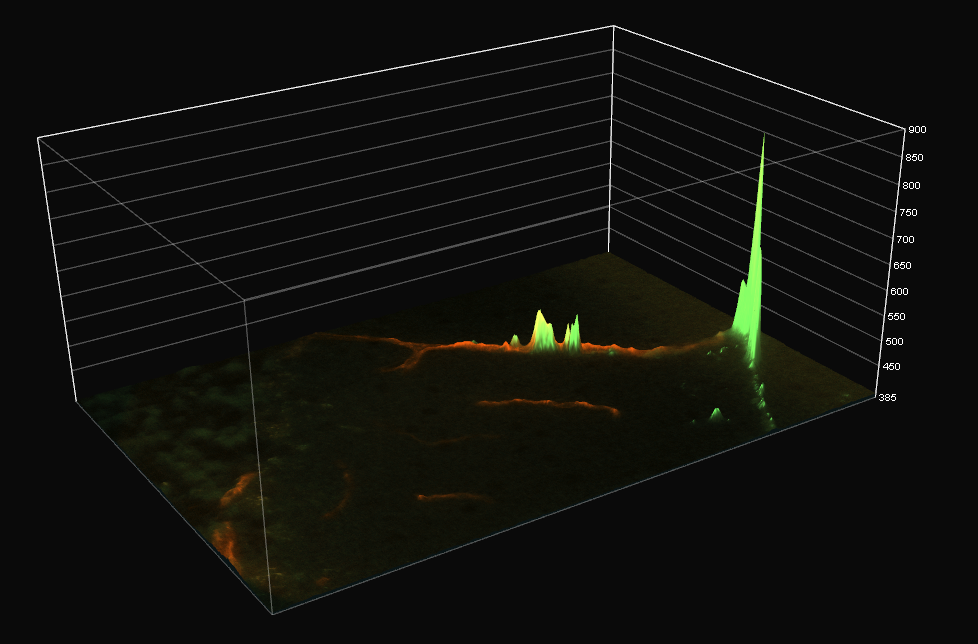


Cerebellum, rat.

Top: 20 μm slice, 0.3X0.2 mm. Red: blood vessels. Green: FITC-hrNAGLU.

Bottom: surface intensity plot for the above image.

F


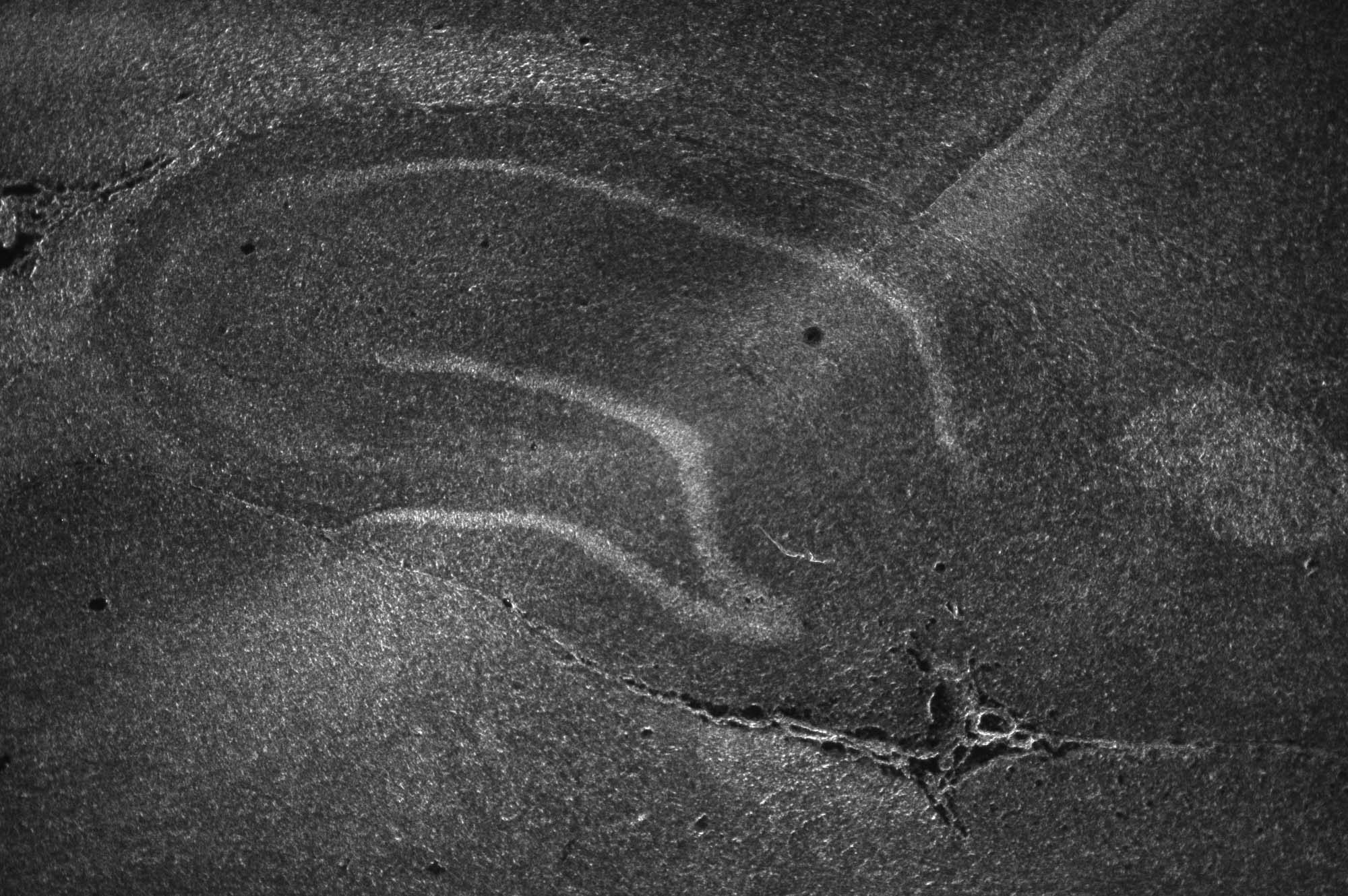


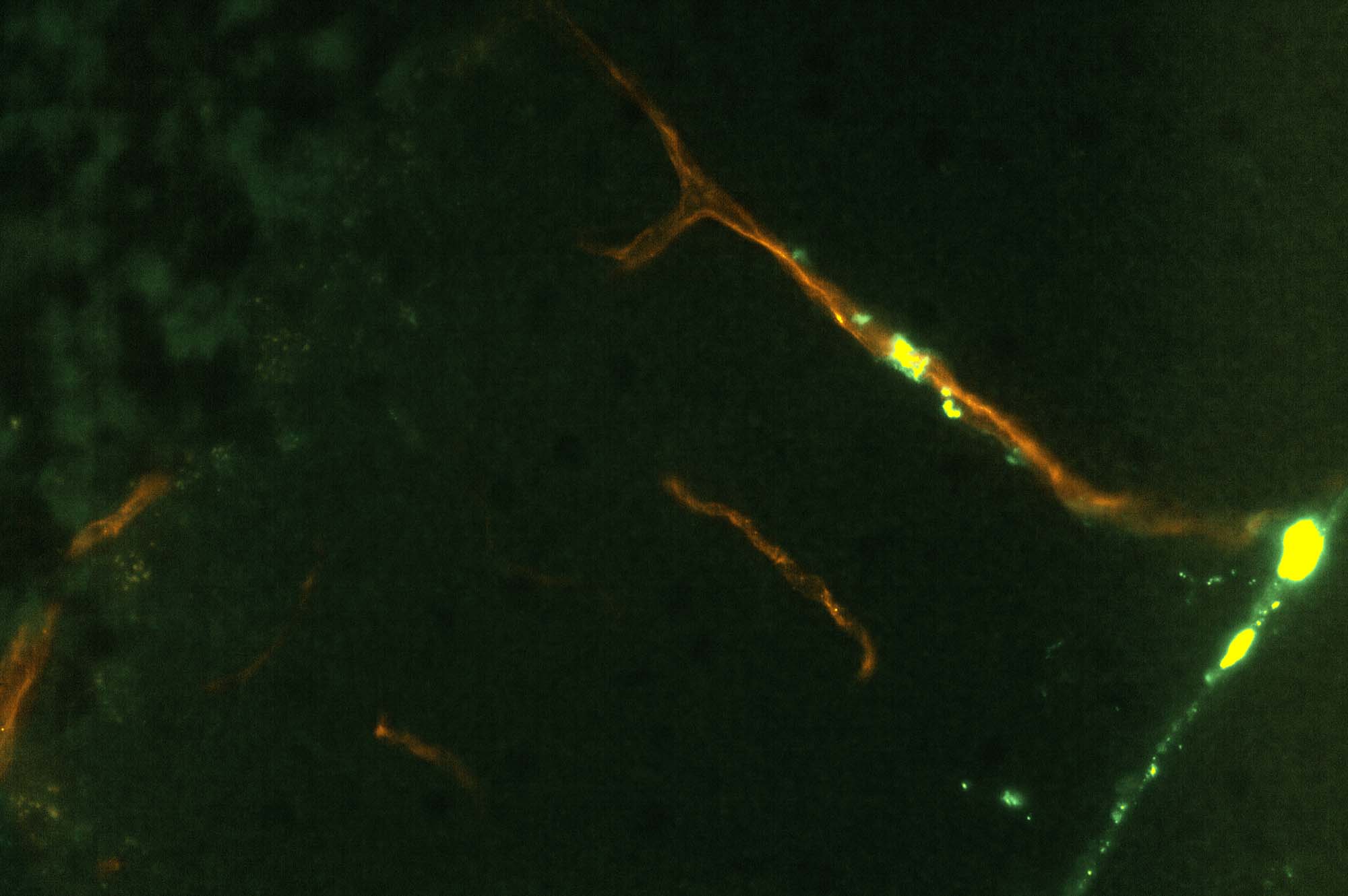


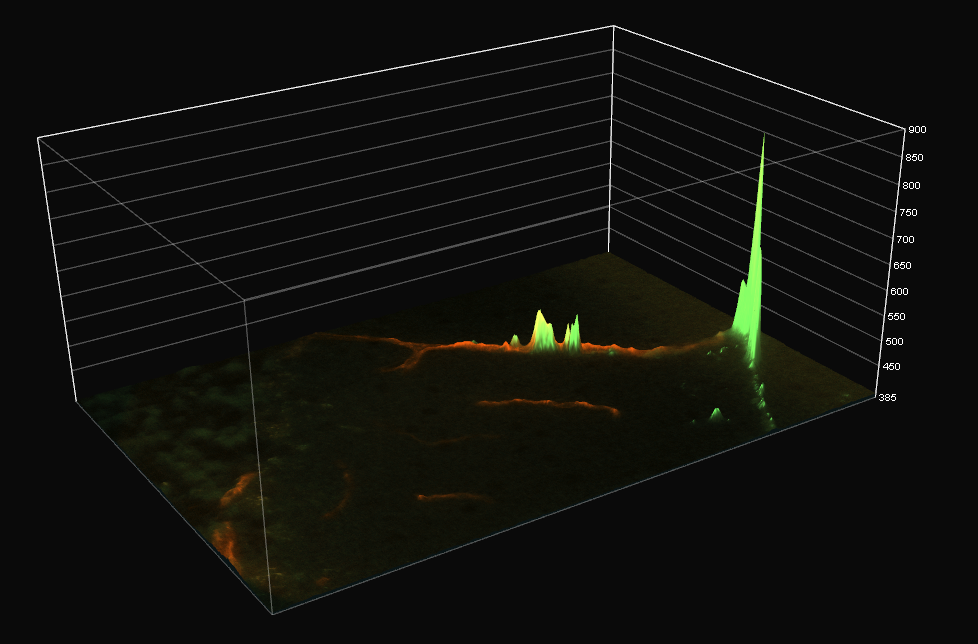


Figure S5. Cerebellum, rat.

Top: 20 μm slice, 0.3X0.2 mm. Red: blood vessels. Green: FITC-hrNAGLU.

Bottom: surface intensity plot for the above image.

a


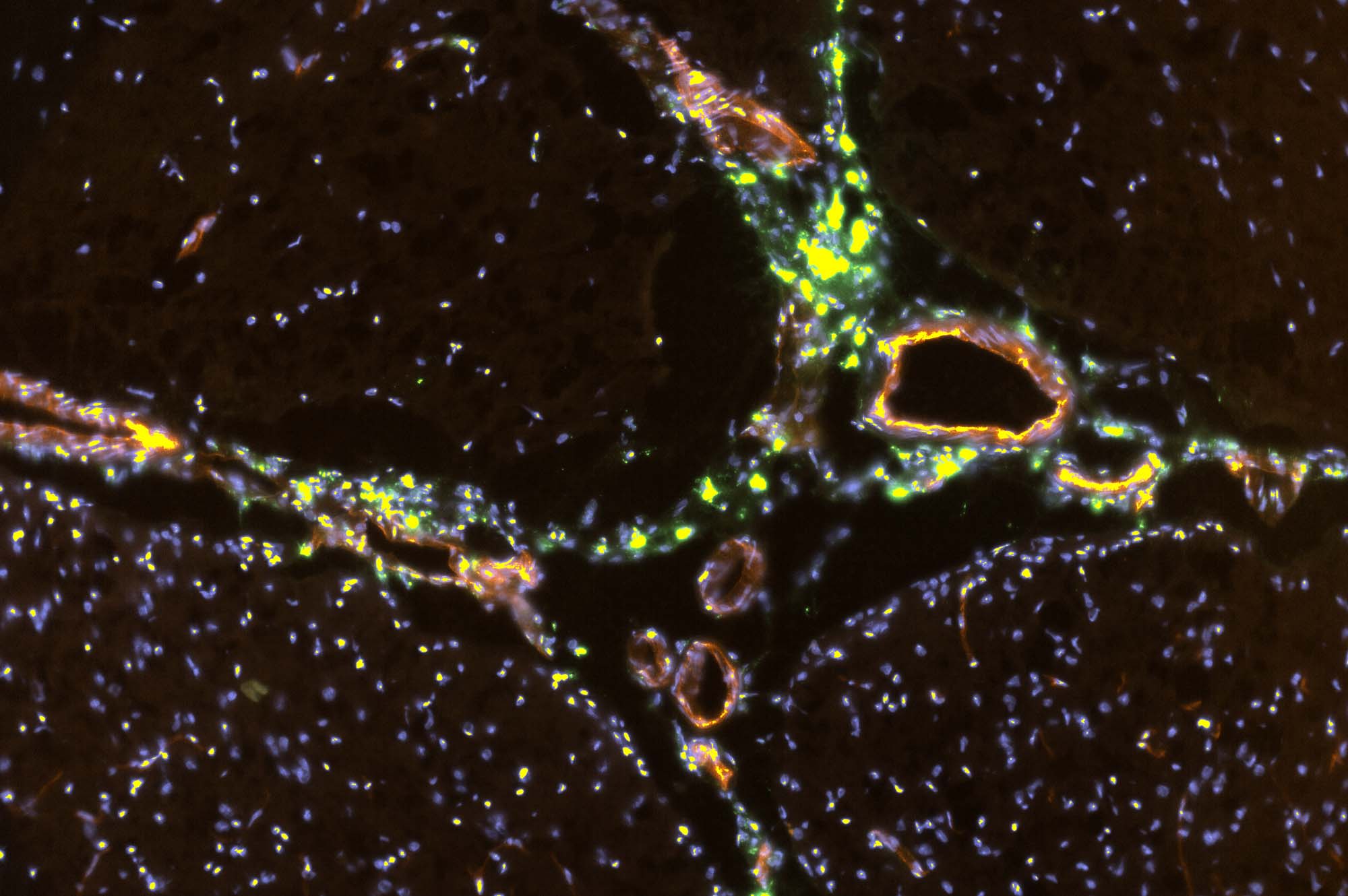


b

Figure S 6

The anatomical context of Figure 2. Rat brain, subhippocampal cistern (subhippocampal extension of the quadrigeminal cistern); arrow.

(a) Brightfield image of a sagittal section, ca. 3 mm off the central plane, of rat brain. Rectangle: area corresponding to fluorescence photoimage in Figure 2 (b).


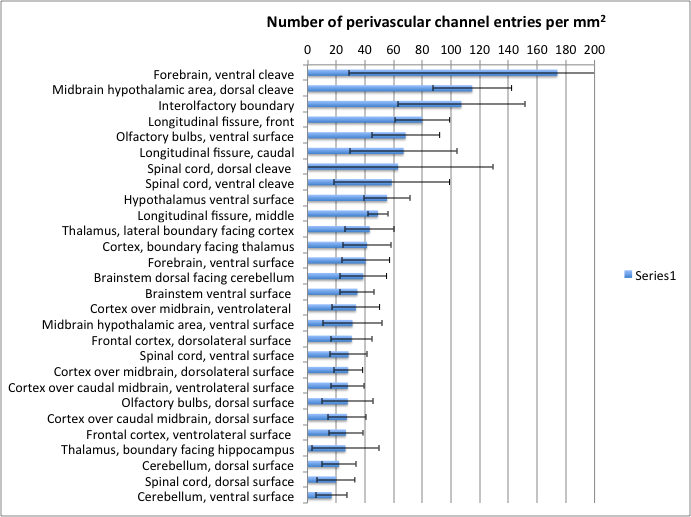


Figure S7. Densities of perivascular channel entries on the boundaries of CNS.


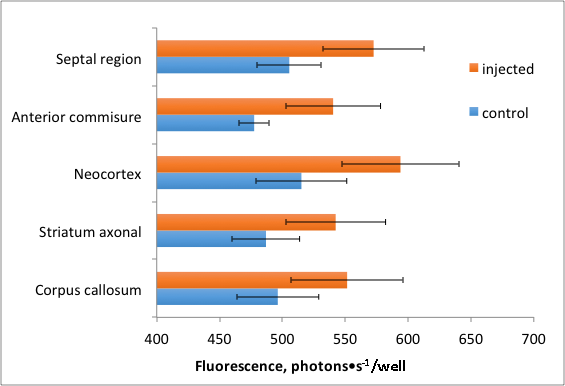


Figure S8. Fluorescence of parenchymal non-perivascular cells after administration of FITC-rhNAGLU to the CSF (rat, 24 hours after the administration).

Figure S9. – high resolution versions of Figure 1 a of the paper.


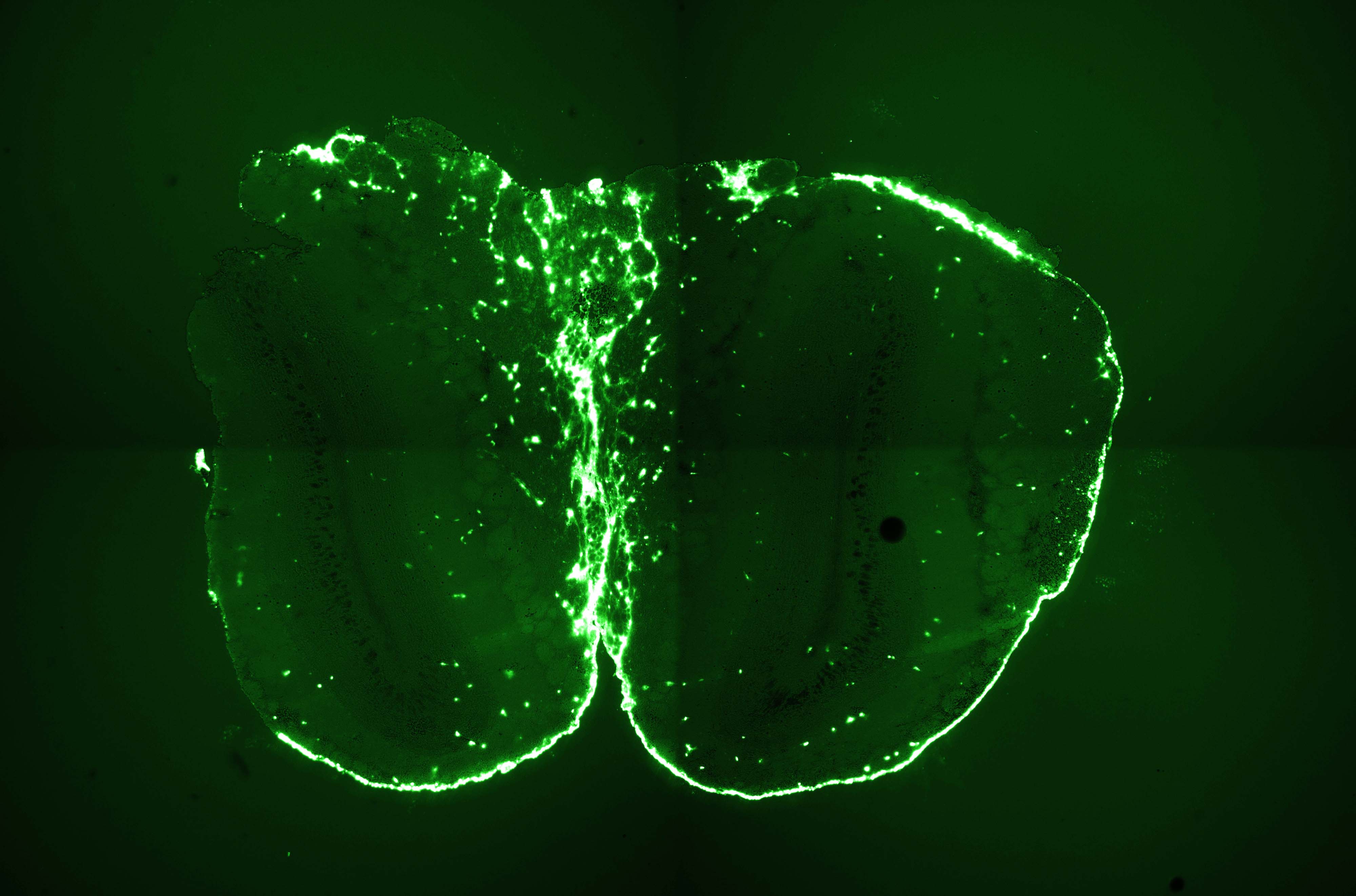


Figure S10. – high resolution versions of Figure 1 b of the paper.


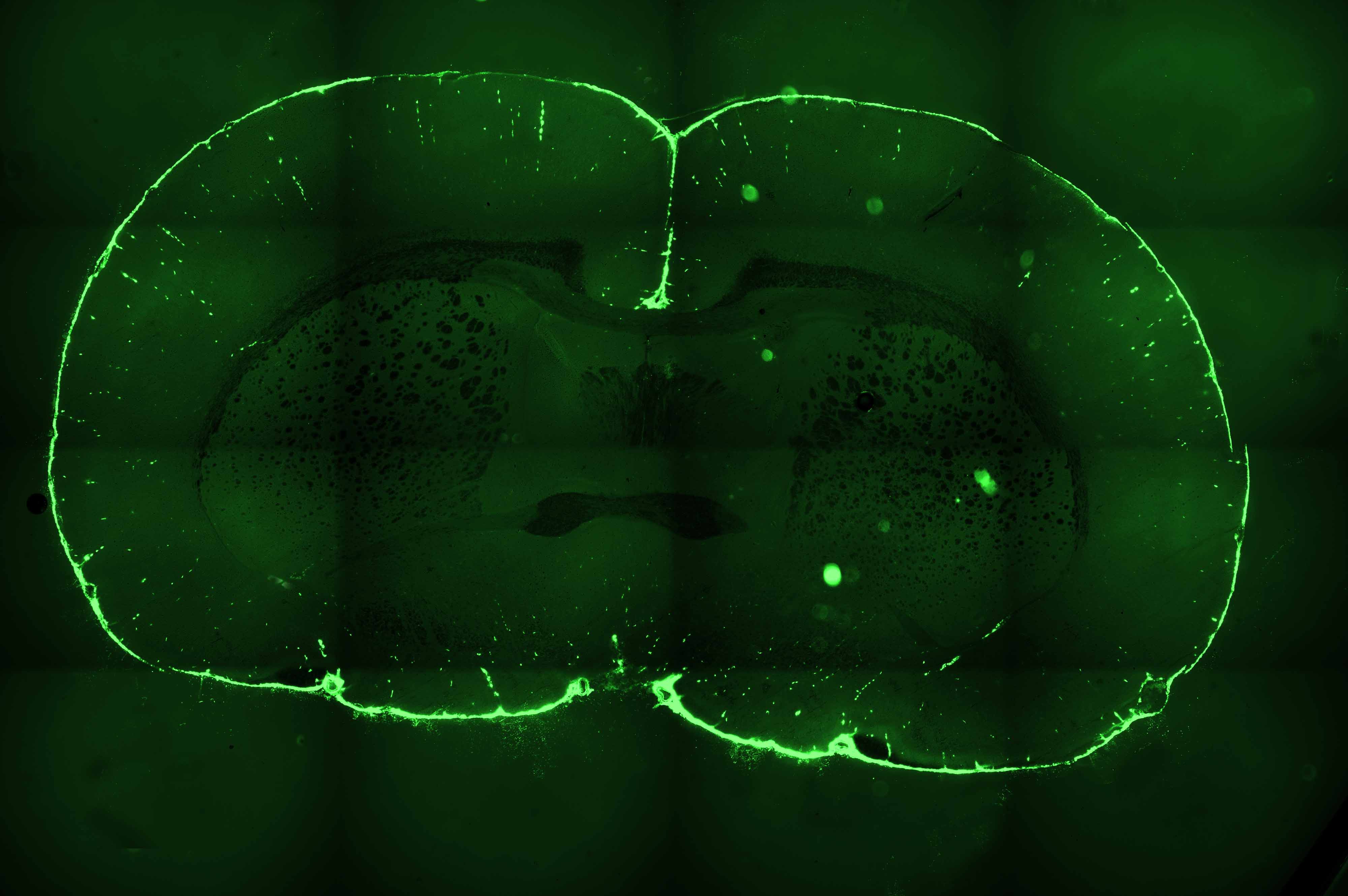


Figure S11. – high resolution versions of Figure 1 c of the paper.


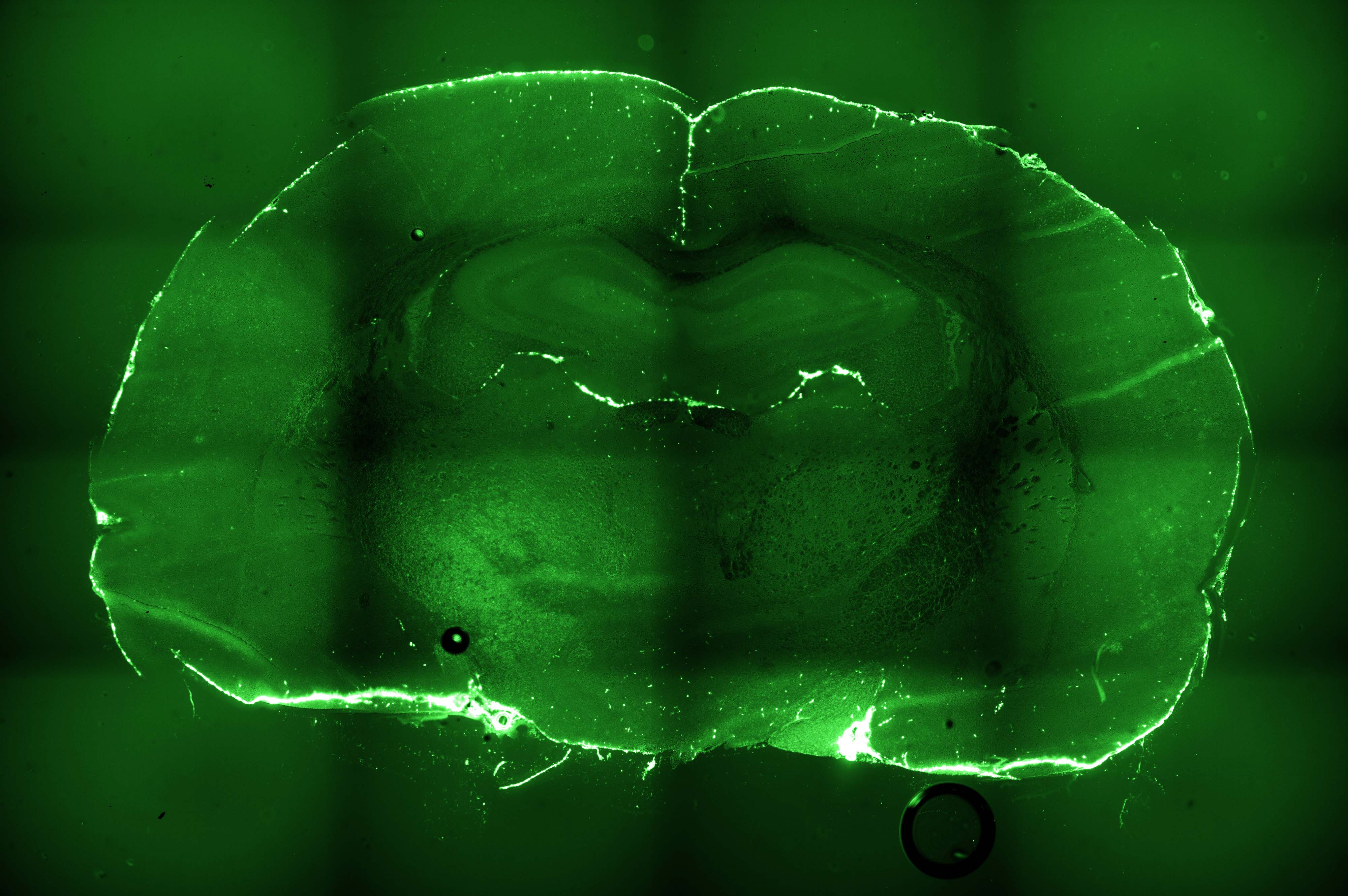


Figure S12. – high resolution versions of Figure 1 d of the paper.


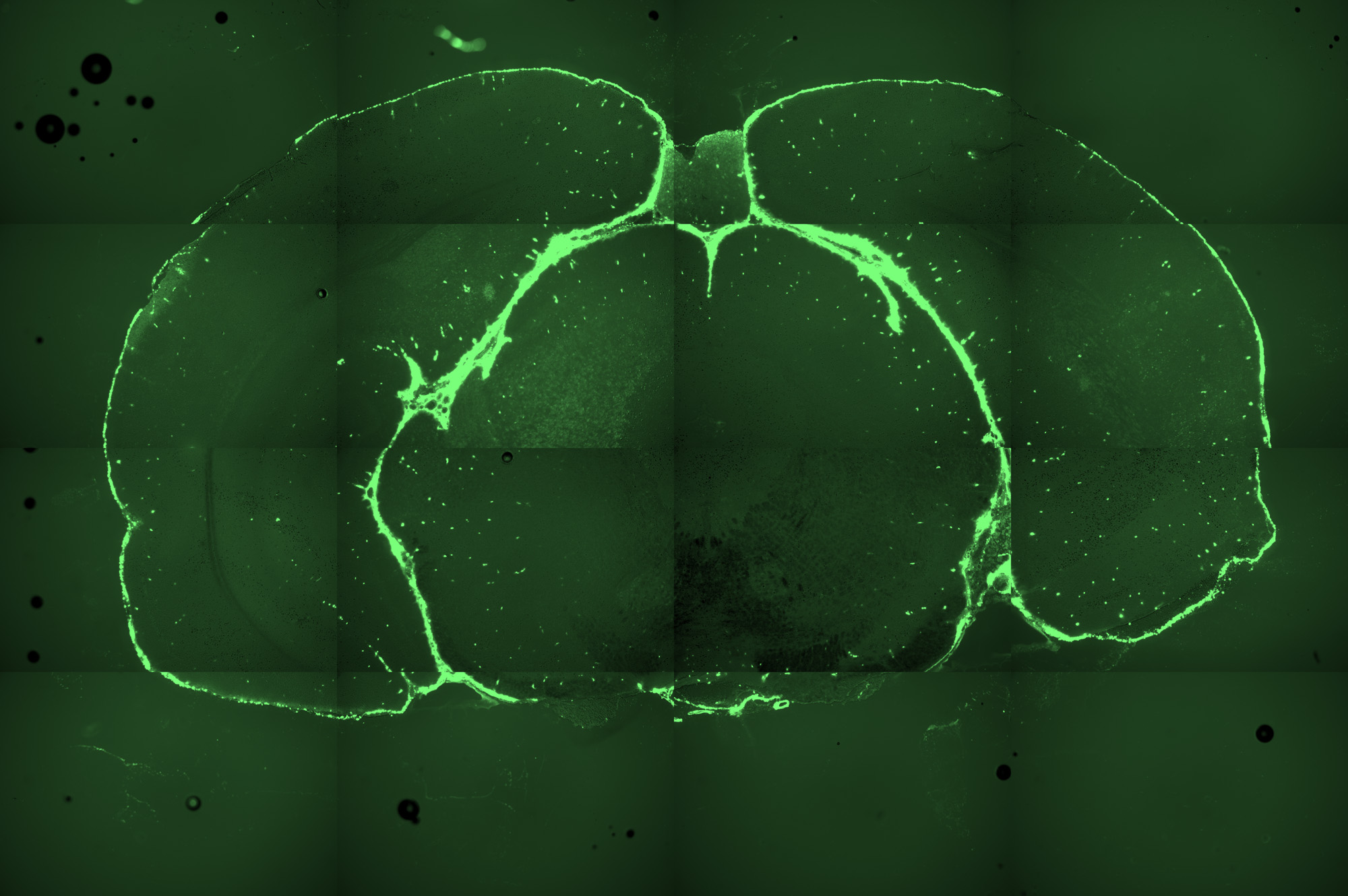


Figure S13. – high resolution versions of Figure 1 e of the paper.


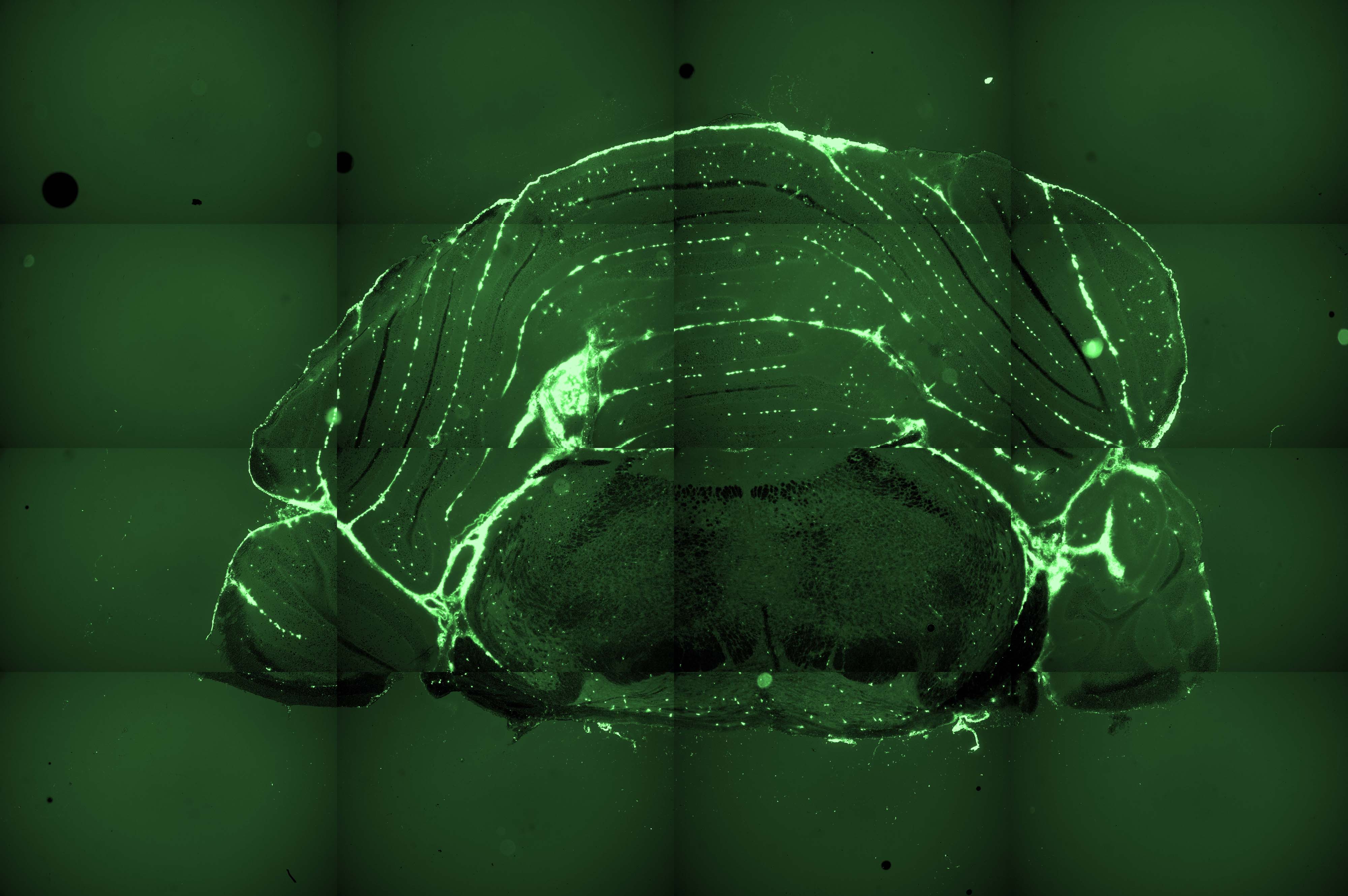


Figure S14. – high resolution versions of Figure 1 f of the paper.


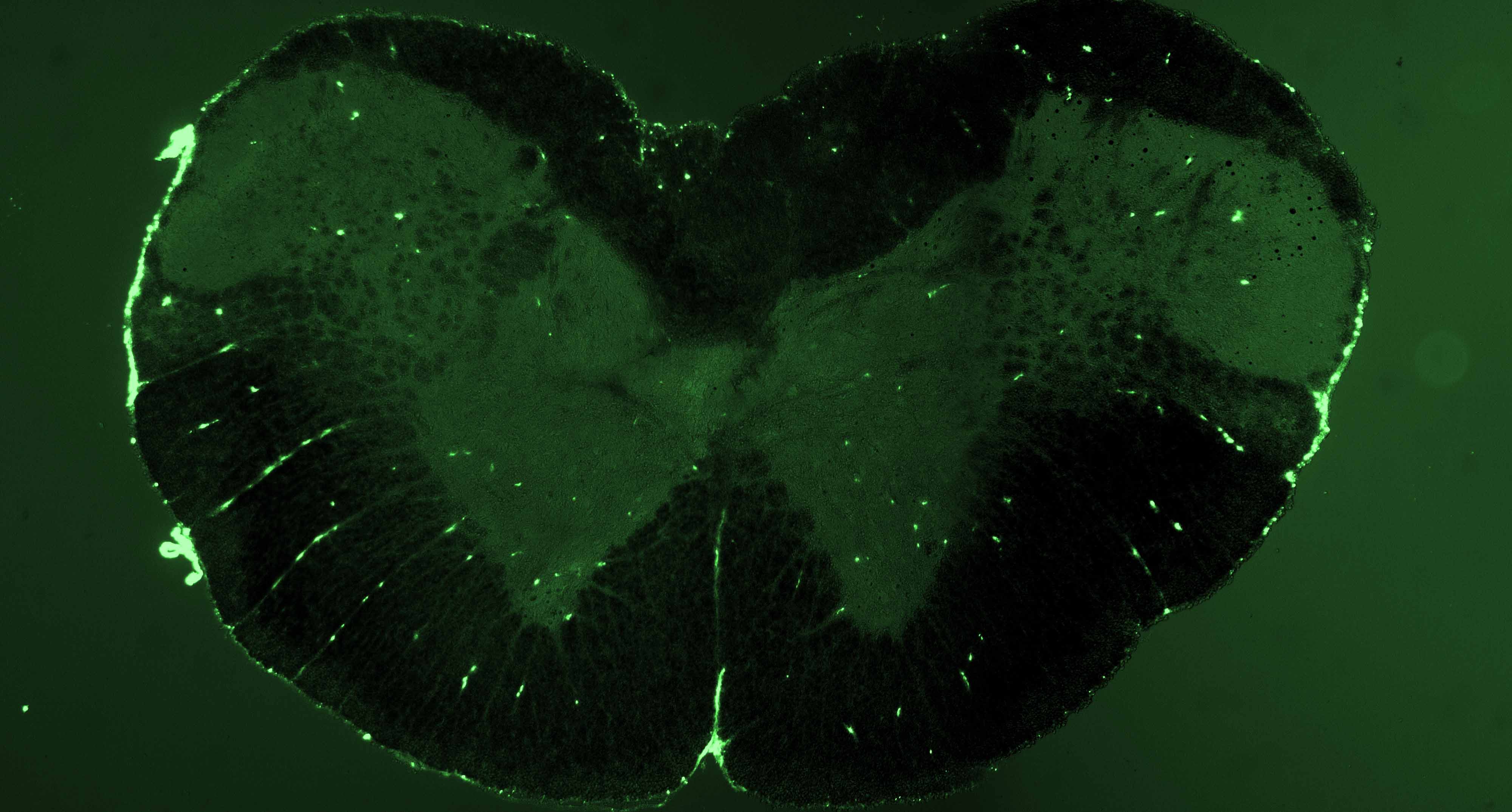


Figure S15. – high resolution versions of Figure 6a of the paper.


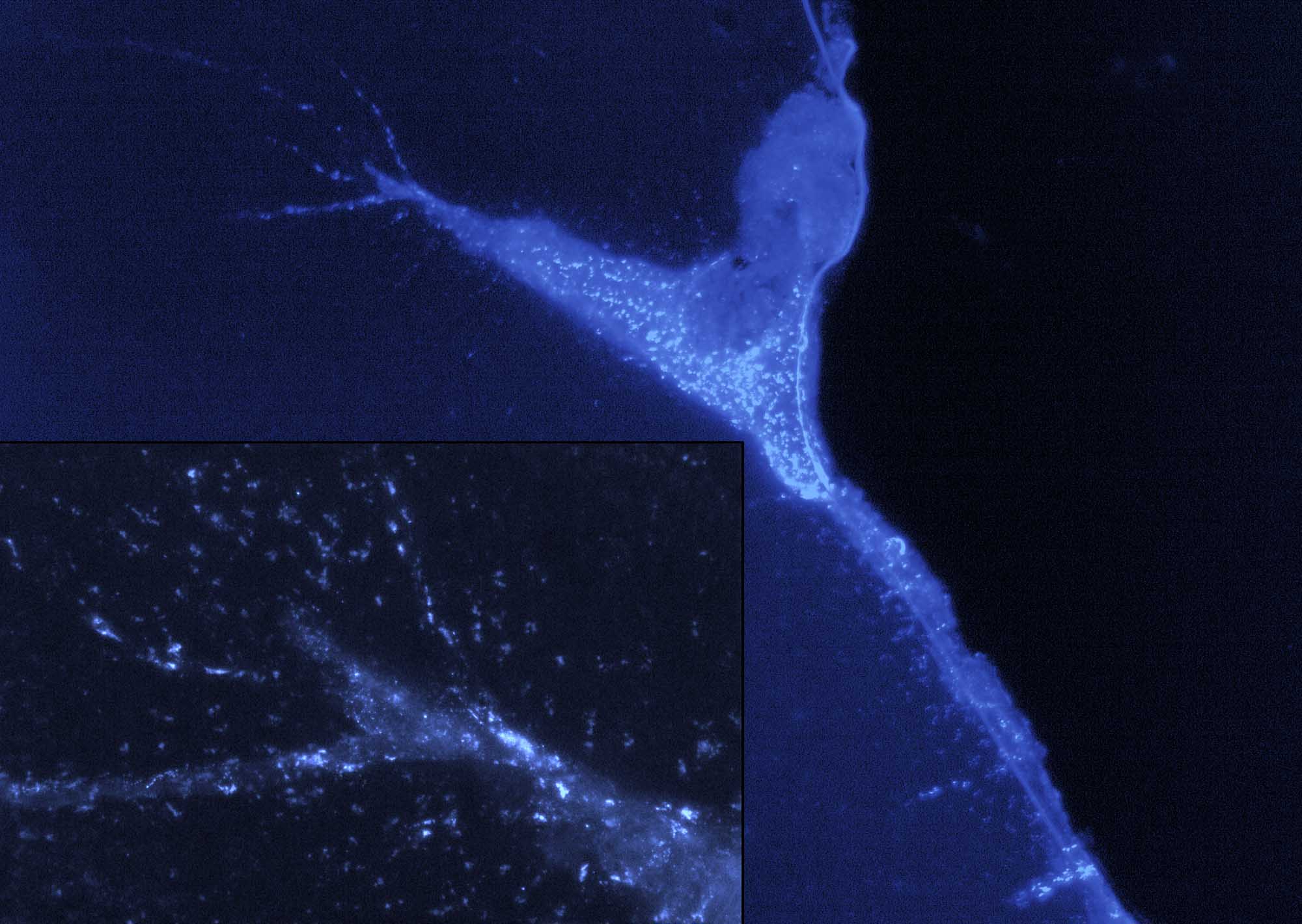


Figure S16. – high resolution versions of Figure 6b of the paper.


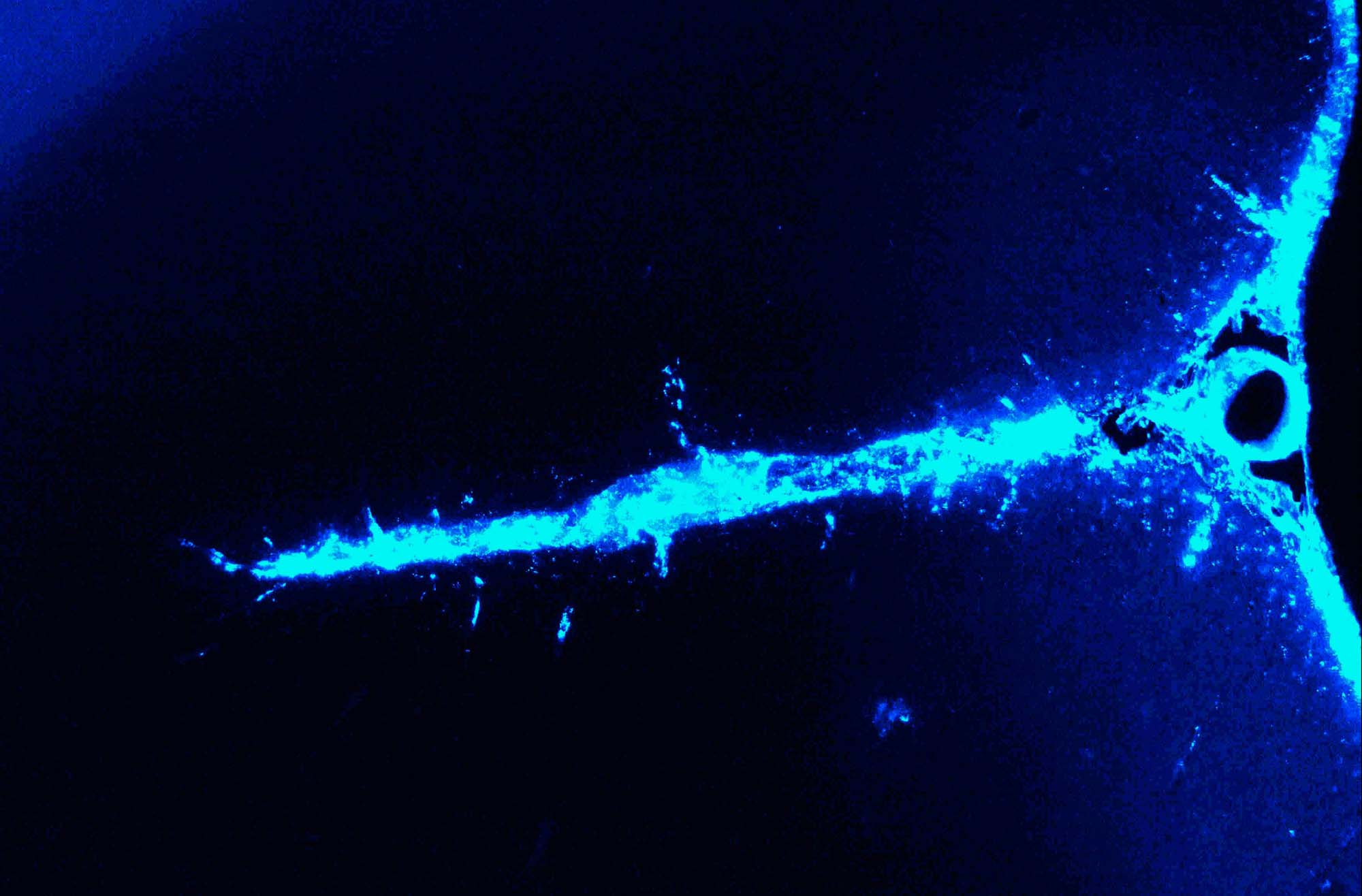


SAS

A

Figure S17. Monkey, 100 μm section, 4.4X2.9 mm.

Perivascular channel branching from the central fissure.


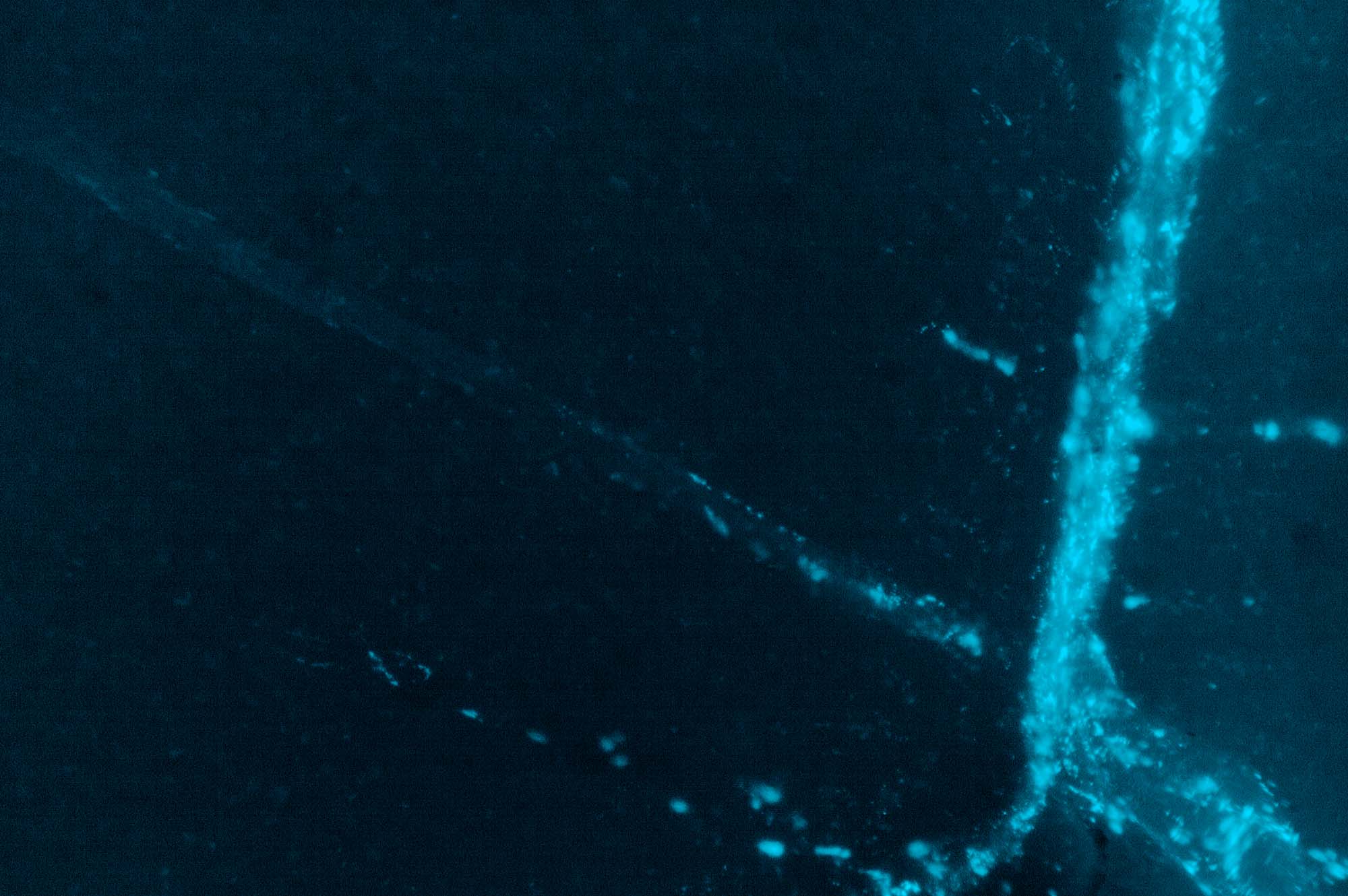


CF
